# Giant panda BAC library construction and assembly of a 650-kb contig spanning major histocompatibility complex class II region

**DOI:** 10.1186/1471-2164-8-315

**Published:** 2007-09-08

**Authors:** Chang-Jun Zeng, Hui-Juan Pan, Shao-Bin Gong, Jian-Qiu Yu, Qiu-Hong Wan, Sheng-Guo Fang

**Affiliations:** 1 College of Life Sciences, Zhejiang University, Hangzhou 310058, P. R. China; 2 State Conservation Center for Gene Resources of Endangered Wildlife and the Key Laboratory of Conservation Genetics and Reproductive Biology for Endangered Wild Animals of the Ministry of Education, Hangzhou 310058, P. R. China

## Abstract

**Background:**

Giant panda is rare and endangered species endemic to China. The low rates of reproductive success and infectious disease resistance have severely hampered the development of captive and wild populations of the giant panda. The major histocompatibility complex (MHC) plays important roles in immune response and reproductive system such as mate choice and mother-fetus bio-compatibility. It is thus essential to understand genetic details of the giant panda MHC. Construction of a bacterial artificial chromosome (BAC) library will provide a new tool for panda genome physical mapping and thus facilitate understanding of panda MHC genes.

**Results:**

A giant panda BAC library consisting of 205,800 clones has been constructed. The average insert size was calculated to be 97 kb based on the examination of 174 randomly selected clones, indicating that the giant panda library contained 6.8-fold genome equivalents. Screening of the library with 16 giant panda PCR primer pairs revealed 6.4 positive clones per locus, in good agreement with an expected 6.8-fold genomic coverage of the library. Based on this BAC library, we constructed a contig map of the giant panda MHC class II region from *BTNL2 *to *DAXX *spanning about 650 kb by a three-step method: (1) PCR-based screening of the BAC library with primers from homologous MHC class II gene loci, end sequences and BAC clone shotgun sequences, (2) DNA sequencing validation of positive clones, and (3) restriction digest fingerprinting verification of inter-clone overlapping.

**Conclusion:**

The identifications of genes and genomic regions of interest are greatly favored by the availability of this giant panda BAC library. The giant panda BAC library thus provides a useful platform for physical mapping, genome sequencing or complex analysis of targeted genomic regions. The 650 kb sequence-ready BAC contig map of the giant panda MHC class II region from *BTNL2 *to *DAXX*, verified by the three-step method, offers a powerful tool for further studies on the giant panda MHC class II genes.

## Background

The giant panda (*Ailuropoda melanoleuca*), as one of most widely recognized conservation icons in the world, was once distributed over southern and eastern China and extended to northern Burma and northern Vietnam. Unfortunately, habitat loss and fragmentation, low genetic diversity and small population size all lead to current endangered status of this rare species. The estimated 1100 giant pandas survive only in a fraction of their historical range, six completely isolated mountain ranges [1,2]. In order to protect this rare species, considerable efforts were made in different research fields. However, the panda genome still remains unknown. One goal of this study is to construct a bacterial artificial chromosome (BAC) library for the giant panda in order to provide a new tool for panda genome physical mapping.

Genes of major histocompatibility complex (MHC) form one of the most important genetic systems for infectious disease resistance in vertebrates [3]. MHC-encoded genes have been demonstrated to be associated with susceptibility to numerous infectious [3], take part in mate choice of vertebrates [4,5] and control the compatibility between mother and fetus during pregnancy [6], making the MHC a research field of considerable biological interest.

The giant panda MHC remains relatively little known. Furthermore, the limited references were published by our research group: (1) the giant panda MHC was located on chromosome 9q by fluorescence in situ hybridization [7]; (2) the levels of genetic variation for the MHC class II *DRB *and *DQA *loci in the giant panda were low, only 7 *DRB *alleles and 6 *DQA *ones survived in current populations [8,9]. On the other hand, it has been reported that the giant pandas are particularly susceptible to infectious disease and parasites, such as 100% for ascariasis and 20% for ticks, resulting in a 66.67% mortality rate from ascariasis [10-12]. As a result, exploring genetic characteristics of multiple MHC loci in the giant panda has become more and more essential. However, the number of MHC genes in the giant panda keeps unknown all the time.

Based on an increasing number of published data from human, Horton *et al*. divided the human MHC into five physically adjacent subregions: extended class I (from *HIST1H2AA *to *MOG*), classical class I (from *C6orf40 *to *MICB*), class III (from *PPIP9 *to *NOTCH4*), classical class II (from *C6orf10 *to *HCG24*), and extended class II (from *COL11A2 *to *RPL12P1*) [13]. The *HLA *class II cluster comprises the classical class II genes (*HLA-DR*, *-DQ *and *-DP*) and the non-classcial class II gene (*HLA-DM *and *-DO*) [13]. Based on multiple suits of MHC data from different mammals, scientists have found that the mammalian MHC classical class II subregion generally contains *DR, DQ, DP, DM *and *DO *genes and the organization of the classical class II (*BTNL2 ~ DR ~ DQ ~ DOB ~ DM ~ DOA ~ DP*) is relatively conserved [14-18]. Additionally, some genes within extended class II subregion, such as *COL11A2 *and *DAXX*, are strongly conserved in vertebrates from bony fish to human in the evolutionary spectrum [19]. Due to complete lack of genomic and mapping resources, the MHC studies for the giant pandas become more tedious and difficult. As a result, another goal of this study is to construct a contig spanning the whole classical class II and part of the extended class II regions (i.e. from *BTNL2 *to *DAXX*) based on the giant panda BAC library.

## Results and discussion

**Figure 1:**
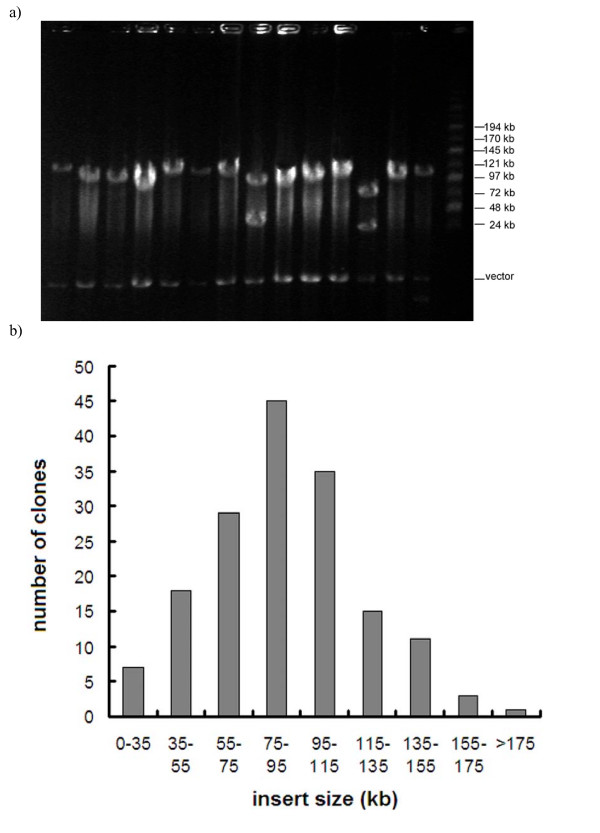
(a) Insert size analysis of randomly selected BAC clones. There are 14 BAC clones tested. The BAC DNA was completely digested with *Not I *enzyme and determined by pulsed field gel electrophoresis with 1–20 second switch time, 6 V/cm at 12°C for 16 hours. The MidRange II PFG markers (New England BioLabs) were used as size standard in the right flanking lane. The size of pCC1BAC vector is 8.1 kb. (b) Distribution of insert size in the giant panda BAC library. Insert sizes were evaluated by PFGE after *Not I *restriction enzyme digestion from 174 BAC clones.

We successfully constructed a deep coverage, large-insert and publicly available *Hind *III BAC library for a female giant panda. The giant panda BAC library consisted of 205,800 clones arrayed in 2,100 96-well plates. To estimate the average insert size of the library, 174 clones were selected randomly and tested by pulse field gel electrophoresis (PFGE) (Figure 1). The insert size was calculated using the TotalLab 1D gel analysis program. The results showed that the average insert size was 97 kb. About of 5.74% non-insert clones were observed in the 174 clones tested (10 out of 174). According to the published data on haploid C values (CV) of the Carnivora [20], assumed a similar genome size with the size of family ursidae, the giant panda BAC library constructed here had 6.8-fold genome equivalents. Theoretically, the probability of any giant panda gene being found in this library should be about 99%.

In order to enable a rapid and efficient screening of the giant panda BAC library by PCR, 205,800 clones were divided into 43 superpools, each consisting of 4704 clones (49 × 96-well plates). In the following steps, 49 plates involved in a superpool were arrayed in a 7 × 7 matrix and 4D-PCRs were performed [21]. A total of 16 giant panda gene loci were utilized in 4D-PCR for coverage testing (Table 1). Two or more positive clones (2 ~ 16) were found for these 16 loci, with an average of 6.4 positive clones per locus (see Table 1). The PCR screening results were thus in agreement with an expected 6.8-fold genomic coverage of the library.

**Table 1:** Library screening results based on PCR amplification of 16 pairs of primers*

Locus	Type	Primer sequences (5'→3')	PCR product size (bp)	Number of positive clones	Source
Notch4	MHC class III	CGCTGTGCCTGCCTCAAT CATCACCTATGCCCTGAC	217	8	GenBank: AY973811
DRA	MHC class II	AGACAAGTTCTCGCCACC CTCCACCTTGCAGTCATAGA	172	2	GenBank: AY973816
*IGHC*	Genomic sequence	CAGCCCTCTTCCTCCTCACTC CGCCCAGCATCTGTTTCCA	244	5	GenBank: AY818395
*MC1R*	Genomic sequence	GCGGGCCATCTCCACTAT TTGAGGGCAGAGGACCAT	322	6	GenBank: AY884206
*DRB*	MHC class II	TCGCGTCCCCACAGCACATT GCCGCTGCACACTGAAACTCTCAC	281	12	[8]
*DQA*	MHC class II	GCTGACCATGTTGCTTACTAT AAGAGGCAGAGCATTGGACA	275	16	[9]
*MHC13*	MHC class I	GCTCCCACTCSMTGAGGTATT CTGCCTCGCTCTGGTTGTAG	293	6	GenBank: DQ367726
*HSP70*	MHC class III	TGCTGATCCAGGTGTACGAG CGTTGGTGATGGTGATCTTG	204	8	GenBank: M69100
*G001*	Micro-satellite	ACGGGAAGCCTGCTTCTACACTC AGACACCCAACCGACTAAACCAC	164–172	6	[22]
*G905*	Micro-satellite	CTGGCTTCAACTGCCTTTGAGAG GCACCTGGATTTGTGATGCTATC	169–177	5	[22]
*Ameμ11*	Micro-satellite	TATGCCACCTGCCCAGAC GATGGAAAGAGTAGAGCCAAGG	248–256	4	[23]
*Ameμ15*	Micro-satellite	AAGCAGTTGTTTTTGCTTAGTG TGTCAAAGTATTTGCCTCACA	142–150	3	[23]
*Ameμ19*	Micro-satellite	CAGGCAGCACAGCTATACCA CCACCTGATACCTATGCACAT	174–182	6	[23]
*Ameμ22*	Micro-satellite	AGGAAACATGTTGCCTTTTCA AGAGGGCAAATAGGAGGGAA	147–149	4	[23]
*Ameμ25*	Micro-satellite	CATAATTCCCTGGCAATGCT TGCCCGCATTGAAAAATG	239–257	3	[23]
*Ameμ26*	Micro-satellite	TTTTCAGGCCTCCGAAAAC ATTCCCAATAAAGCAAATCAGA	134–140	5	[23]

*To avoid false-positive results, individual positive clones were verified by the second round PCR. All the PCR products of positive clones were sequenced three times.

Compared the giant panda BAC library with other mammalian BAC libraries [24-27], the average insert size of the panda BAC library was relatively small. In this study, we separated the partial digestion HMW DNA with two size-fractionated methods as described by Osoegawa *et al*. [28] and further removed the trapped small fragment by concentration with modification procedures. In general, difficulties in cloning are encountered when attempting to increase the size of the cloned fragments. Removing small restriction fragments is vital for constructing a high-quality BAC genomic library. Although the small fragments could be eliminated more efficiently by PFGE, small DNA fragments are subject to remaining trapped in or co-migrated with the desirable-size fragments [29]. Hence, the primary reasons of large DNA molecules unable to be cloned into BAC vectors are probably due to damage to larger DNA molecules during the process of preparation and purification and subsequent preferential cloning of smaller DNA molecules [30].

In order to construct a BAC clone-based contig map of giant panda MHC class II region between *BTNL2 *and *DAXX *loci, we designed ten sets of MHC primers based on homologous class II MHC genes [15,16,31] and partial MHC sequences of giant pandas (AY973813-16), which were from *BTNL2*, *DRA*, *DRB*, *DQA*, *DOB*, *LMP2*, *DMB*, *DMA*, *COL11A2*, and *DAXX *genes, respectively. All the positive BAC clones were end-sequenced for designing primers but some ones failed to implement library screening. Two BAC clones (692B2 and 1262B6) were subject to shotgun sequencing to recruit new end sequences. All the sequences were analyzed first using RepeatMasker [32] in order to avoid that repetitive elements were designed as primers, which is of great importance for subsequent library screening. Similarly, only gene-specific primerpairs could be employed to verify overlapping between BAC clones. In the present study, the ten suits of gene-located primers were all from unique regions as shown in mammals such as *BTNL2*, *DOB*, *LMP2*, *DMB*, *DMA*, *COL11A2 *and *DAXX*, or from single loci as validated by population investigation like *DRA *[unpublished data], *DRB *[8] and *DQA *[9]. Therefore, these specific primers will help to assemble the BAC contig map of MHC class II region without confusing.

Using above-mentioned locus-specific primers (Table 2), we screened the giant panda BAC library and obtained a number of PCR positive BAC clones. The subsequent sequencing results showed that the amplified sequences from genes *BTNL2*, *DOB*, *LMP2*, *DMB*, *DMA*, *COL11A2*, *DAXX *and *DRA *were identical, suggestive of homozygotes at these loci. Differently, the primerpairs of *DRB *and *DQA *both obtained two different sequences, indicative of heterozygotes at these two loci. Despite the library coverage testing revealed that the numbers of positive *DRB *and *DQA *clones were 12 and 16, respectively (Table 1), the number of *DRB *or *DQA *alleles was only two for these positive BACs, consistent with the characteristics of one locus as described previously [8,9]. Furthermore, the two *DRB *alleles and the two *DQA *ones in this study were not new but repeated previous alleles reported by Wan *et al*. [8] and by Zhu *et al*. [9], demonstrating that both *DRB *and *DRA *primerpairs were able to exclusively amplify one locus. However, caution is required when asserting that the giant panda possesses only one *DRB *or only one *DQA*. In our pre-experiments we employed other two sets of exon 3-located *DRB *and *DQA *primers (in this case exon 2-located) and both obtained about 20 positive BAC clones, presenting three and four different sequences for the *DRB *and *DQA *primers, respectively. The results of pre-experiments indicated that the giant panda probably had not less than one *DRB *or one *DQA *locus. Considering the requirement of correct contig assembly and inter-loci confusion of the exon 3-located primers, we finally abandoned these two suits of exon 3-located primers and adopted the two exon 2-located primerpairs, which had been proven to be one-locus-amplified in our colleagues' studies [8,9].

**Table 2:** Primer list used for constructing the final contig with minimum tiling path

Primer name	Source	Primer sequences (5'→3')	Size (bp)	GenBank No.
*BTNL2*	GenBank: AY152836.1 GenBank: AJ630362	GGGAAGATGCTCTACTCA GATGGTCGAATGTTGTTT	223	EF125957
*DRA*	GenBank: AY973816	AGACAAGTTCTCGCCACC CTCCACCTTGCAGTCATAGA	172	EF125964
*DRB*	[8]	TTCACCAACGGGACGGAG GCTGCTGCTCCATGAAGTC	281	AY895156
*DQA*	[9]	CTTACCCTACTGACCAGC TAAAGAGGCAGAGCATT	275	EF664077
*DOB*	GenBank: AY973815	GGGGACAGTGTATCCAGAG AGGAGTCATTTCCAGCATC	202	EF125962
*LMP2*	GenBank: AY152832.1 GenBank: AJ630364.1	CCCACTAGAGCCATCCCG CAGCCCTTTGCCATCGGT	703	EF125966
*DMA*	GenBank: AY973813	GAAGCCCCTGGAGTTTGG GGCCTGGAAGCTGAGTTCAT	157	EF125960
*DMB*	GenBank: AY973814	GCTTCTATCCAGCTGATG GGTGTAAGTGTCCCCGTA	155	EF125961
*COL11A2*	GenBank: AY957499.1 GenBank: AJ630366.1	CTCAAACACTTCTTCATCCAG GACCAGGTGGCACCGTGT	168	EF125958
*DAXX*	GenBank: AJ630366.1 GenBank: AY152827.1 GenBank: BC109074.1	TCGCTCCTGTAACCTGATG CTCTTTGGGCGGCTTTGT	282	EF125959
237D4-T7	End sequence	AGTTCTCCATCCGGCTCT TACCATCCTGGTGTCAGT	172	/
237D4-RP2	End sequence	TTGGAAAGTCTGGGATGA TGTGCTCTGGAAAGATGTG	274	/
826G2-T7	End sequence	CTCCCTCAGCAATAACC CAAAGAGTGAACCTGCAAAACC	187	/
826G2-RP2	End sequence	TGTGACCGAGTTGGAGTA GAAAGAGAGATGGGGATG	442	/
900H8-T7	End sequence	GAAGTGAGGGTAGAGGAC AAGCTGCTGTTAGAAATG	249	/
206G5-T7	End sequence	AGAGGTCACGACCAGCAC TTTGGAAGGTTGTTTATCAC	207	/
692B2-RP2	End sequence	AGCACGGCCATAATAATAAA GAGGGACAAGGTTGGACAG	264	/
692B2-F8R8	Shotgun sequences	TCCTTTAGCCCCTCTTGAGCC CCAACCCGCGTGTACTTTTGG	195	/
1262B6-F5R5	Shotgun sequences	CCTCCACATTTCTCTGCTT GCCATGGGTATAGTTACCT	273	/
186A7-T7	End sequence	GCCTCTTGTCCAGTTCCT GGTCTCCCTCTGTTGCTC	198	/

**Figure 2:**
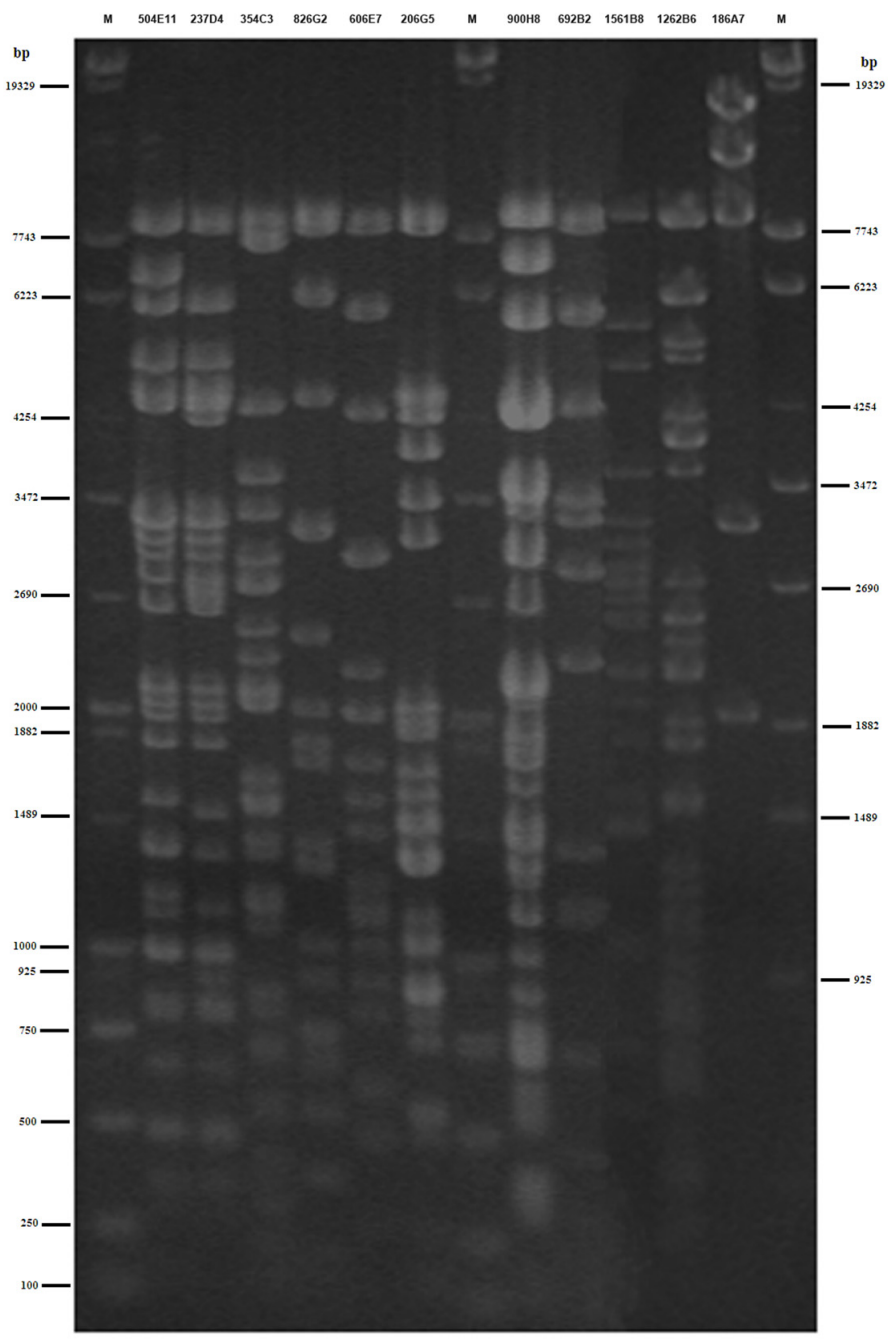
DNA fingerprints of 11 positive BAC clones involved in minimum tiling path. The positive BAC clones were extracted by Qiagen large-construct kit and digested with *Hind *III and *EcoR *I, followed by separation on 1.0% agarose gel with 2.0 V/cm for 18 hours. M is mixture of λ-EcoT14 I digest and DL2000 DNA marker. The sizes in base pairs of markers are indicated on both sides.

**Figure 3:**
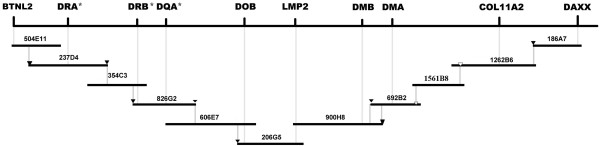
The minimum BAC tiling pattern of giant panda MHC class II region. A total of 11 BAC clones were involved: 504E11 (67 kb), 237D4 (84 kb), 354C3 (69 kb), 826G2 (50 kb), 606E7 (110 kb), 206G5 (73 kb), 900H8 (85 kb), 692B2 (35 kb), 1561B8 (45 kb), 1262B6 (110 kb) and 186A7 (54 kb). Ten MHC class II primerpairs (*BTNL2*, *DRA*, *DRB*, *DQA*, *DOB*, *LMP2*, *DMB*, *DMA*, *COL11A2 *and *DAXX*), eight end primerpairs marked with black triangle (237D4-T7, 237D4-RP2, 826G2-T7, 826G2-RP2, 206G5-T7, 900H8-T7, 692B2-RP2 and 186A7-T7) and two shotgun-sequence primerpairs (692B2-F8R8 and 1262B6-F5R5) marked with blank triangle were anchored to the contig. This BAC contig map of panda MHC class II region spans about 650 kb. The location of genes was determined against the human, dog, cat and other mammalian MHC class II loci. Star symbols indicate the MHC genes are probably multiple loci but this study utilizes one-locus-amplified primers.

Based on the screening results of locus-specific primers, we obtained multiple BAC clusters of genes. Each BAC clone was subject to end-sequencing and primer design in order to analyze BAC overlapping and achieve a minimum tiling path of the panda MHC class II region. As for the BACs were connected simultaneously to the flanking BAC clones, the minimal overlapping was acquired by comparing DNA fingerprinting patterns. According to the results of amplification of end-specific primers (some primerpairs were listed in Table 2), subsequent sequencing and DNA fingerprinting, we finally achieved the minimum tiling path of the panda MHC class II region, which was composed of 11 BAC clones (504E11, 237D4, 354C3, 826G2, 606E7, 206G5, 900H8, 692B2, 1561B8, 1262B6 and 186A7) and whose 11 clone members were all fingerprinted again for ultimate comparison (Figure 2). The minimal tiling path was depicted in a contig spanning about 650 kb (Figure 3), which shows all identified clones with minimized inter-clone overlapping for the giant panda MHC class II region. Although it seems that no obvious overlapping is present among the clones 692B2, 1561B8, 1262B6 and 186A7 in figure 2, their clone connections based on BAC end primers were clearly reflected on figure 3.

Comparisons of the MHC class II region of human *HLA *with dog *DLA *and cat *FLA *showed that the region from *BTNL2 *to *DAXX *was relatively conserved [15,16,31], having a similar organization: *BTNL2 ~ DR ~ DQ ~ DOB ~ DM ~ DOA ~ DP ~ COL11A2 ~ DAXX*. Nonetheless, both *DLA *and *FLA *lost some genes and shrank the *DP *genes into small pseudogenes [15,31], resulting in the regions between *BTNL2 *and *DAXX *of *DLA *and *FLA *were shortened from 928 kb of *HLA *to 669 kb and 675 kb (data from GenBank), respectively. The giant panda MHC class II region presented not only arrangement characteristic of mammalian MHC class II loci; containing *BTNL2*, *DR*, *DQ*, *DO*, *DM*, *COL11A2*, and *DAXX *(Figure 3), but also shortening feature of carnivore MHC class II; being approximately 650 kb long. Worthy of pointing out, we designed multiple sets of primers for both *DPA *and *DPB *based on the homologous counterparts of human, dog and cat but all failed to amplify the *DP *genes (*DPA *and *DPB*), suggesting that the giant panda probably possesses highly variable *DP *genes (such as shrunken ones like those in the dog and cat) or completely lacks *DP *region. For example, cattle, goat and sheep possess ruminant-specific *DY *(*DYA *and *DYB*) instead of *DP *(*DPA *and *DPB*) [31,32]. As a consequence, the structure, organization, amount, and exact order of giant panda MHC class II genes will be unavailable until the contig is sequenced finally.

## Conclusion

The present study reported the construction and characterization of a 6.8-fold giant panda genomic BAC library with an average insert size of 97 kb. The library has been demonstrated to be of good quality by the isolation of multiple BAC clones containing 16 known genes. The giant panda BAC library thus provides a useful platform for physical mapping, genome sequencing or complex analysis of targeted genomic regions. The giant panda MHC class II region was relatively conserved with the counterparts of human, dog, cat and other mammalian MHC class II region. The 650 kb sequence-ready BAC contig map of the giant panda MHC class II region from *BTNL2 *to *DAXX *offers a powerful tool for further studies on the giant panda MHC class II genes. Consequently, we hope that the research works reported here could accelerate different aspects of giant panda studies in order to protect this rare species more effectively.

## Methods

### Library construction

The BAC library was constructed following a previous protocol [28] using the copy-control pCC1BAC vector (Epicentre, Madison, USA). Transformation of the ligation products was performed using TransforMax EPI300 Electrocompetent *E. coli *cells (Epicentre, Madison, USA). A gene pulser II apparatus (Bio-Rad, Hercules, USA) was used and the applied conditions were as follows: voltage 0.9–1.7 kV, resistance 100 ohms, and impedance 25 μF for a 2.5 ms pulse in a 0.1 cm disposable cuvette (Bio-Rad, Hercules, USA). Note that difference in field strength was used for eventually enriching the library. The giant panda BAC library was arrayed into 43 superpools (49 × 96 clones) and screened using a 4D-PCR method [21].

### Preparation high-molecular-weight (HMW) DNA

Whole blood was collected from giant pandas and stored in heparinized sterilized tubes. The lymphocytes cells were harvested by centrifugation and resuspended in ice-cold phosphate-buffered saline (PBS). An equal volume of liquefied (50°C) 2% certified low melt agarose (Bio-Rad, Hercules, USA) was mixed with the cells suspension (~1 × 10^8 ^cells/ml), and the whole mixture was poured into a disposable plug mold (Bio-Rad, Hercules, USA). The following treatments were conducted as described by Osoegawa *et al*. [28]. The DNA plugs were stored in 0.5 M EDTA at 4°C for use.

### Partial restriction digestion of the giant panda HMW DNA with Hind III

Five plugs were equilibrated twice at 4°C with sterilized 0.5 × TE buffer (pH 8.0), each for 1 hour. Then each plug was incubated with 400 μl *Hind *III (TaKaRa) reaction buffer (1 × M buffer, 100 μg/ml BSA, 4 mM spermidine), on ice for 30 minutes. Subsequently, 3.6 units of *Hind *III per DNA plug was added and incubated on ice for 20 minutes to diffuse completely into plug. Partial restriction digestion was carried out by incubating the reaction mixture in a 37°C water bath for 20 minutes. The reaction was stopped by the addition of 1/10 volume of 0.5 M EDTA, pH 8.0. Partially digested giant panda DNA was separated according to a previous protocol [28] with some modifications. Size-fractionated DNA with 150 to 300 kb size range were further concentrated at 4 V/cm, 5 second pulse time at 12°C for 10 hours and thus the trapped small fragments were removed. The sliced agarose plugs containing DNA fragments were placed into dialysis membranes (Spectrum laboratories, Rancho Dominguez, USA) and large DNA molecules were retrieved by electroelution under the condition of 6 V/cm with 30 second pulse time at 12°C for 3 hours.

### Insert size analysis

To analyze the size of insert DNA fragments in this library, 174 randomly selected clones were grown in 10 ml LB containing 12.5 μg/ml chloramphenicol, and the BAC DNA was isolated by alkaline lysis [33]. To avoid contamination of *E. coli *genomic DNA, the DNA was digested with plasmid-safe ATP-dependent DNase (Epicentre, Madison, USA) at 37°C for 2 hours. The purified DNA was digested overnight at 37°C with 25 units of *Not I*. Pulse field gel electrophoresis (PFGE) was performed at 14°C for 16 hours using 6.0 V/cm with 1 – 40 second switch time. Mid-range PFG marker (New England Biolabs) was used as DNA size marker. The gel was stained with ethidium bromide and photographed.

### Coverage testing

For evaluating and testing the coverage rate of this BAC genomic library, PCR-based library screening was performed [21] using 16 giant panda PCR primers (Table 1). For each superpool, seven 1D-PCR and seven 2D-PCR reactions were carried out first to ascertain which 96-well plate contains the target sequence. Then, eight 3D-PCR and twelve 4D-PCR reactions were conducted to find out which BAC clone the target sequence was located in. Finally, each positive clone pre-identified in 4D-PCR was verified individually by the second PCR using the same primer set to avoid false positive results during the process of screening superpools. All the PCR products of positive clones were sequenced three times.

### Routine-, shotgun- and end-sequencing

Conventional PCR products were ligated into pMD18-T vector (Takara) and transformed with DH5α competent *E. coli *cells (Takara). The shotgun library was constructed from BAC DNA and mini-preparation was conducted according to previous protocols [15]. For end sequencing, the positive clones were cultured in LB plus 12.5 μg/ml chloramphenicol and BAC DNA were extracted with Axyprep plasmid miniprep kit (Axygen, CA, USA). End sequencing of the BAC clones was performed on an ABI 377 automated DNA sequencer using pCC1BAC vector-derived sequencing primers: T7 forward (5'-TAA TAC GAC TCA CTA TAG-3') and pCC1/pEpiFOS RP-2 reverse (5'-TAC GCC AAG CTA TTT AGG TGA GA-3'). Routine- and end-sequencing were all performed on a LI-COR 4200 DNA sequencer with sequiTherm EXCEL II DNA sequencing kit (Epicentre Madison, USA).

### Primer design based on homologous, end- and shotgun-sequences

Locus-specific primers were based on homologous sequences from GenBank (Table 1 and 2). The end sequences were analyzed by RepeatMasker [30] to exclude repetitive elements. The end-primers were designed from the BAC end sequences generated using the T7 and RP-2 vector primers. However, because (1) some ends failed to obtain nucleotide sequences; (2) some end sequences were unable to design suitable primerpairs; (3) some end primers screened BAC library unsuccessfully, two BAC clones (692B2 and 1262B6) underwent shotgun sequencing to design new primers adjacent to ends. All the end primers were utilized to amplify the BAC clones and construct the contig by defining overlapping with other BAC clones. End primers of the growing sub-contigs were designed again with primer premier software (version 5.0) and used to further screen the positive BACs to identify new overlapping clones until the gap was filled.

### DNA fingerprinting and contig assembly

The BAC DNA, extracted from MHC positive clones using QIAGEN Large Construct Kit (Qiagen, CA, USA) that can avoid *E. coli *genomic contamination, was fingerprinted by restriction enzyme digestion with *Hind *III and *EcoR *I. DNA fragments were separated on 1% agarose gel with 2 V/cm for 18 hours in 1 × TAE buffer. Restriction fragment patterns were visually compared to ascertain the extent of overlapping between adjacent clones. Finally, 11 BAC clones with minimized overlap were chosen and manually assembled into a contig with minimal tiling path.

## Authors' contributions

CJZ, HJP and SBG performed the experiments and CJZ drafted the manuscript. QHW and SGF provided supervision and revised the manuscript. JQY coordinated the project. All authors read and approved the final manuscript.
